# Multi-Modal Decentralized Hybrid Learning for Early Parkinson’s Detection Using Voice Biomarkers and Contrastive Speech Embeddings

**DOI:** 10.3390/s25226959

**Published:** 2025-11-14

**Authors:** Khaled M. Alhawiti

**Affiliations:** Faculty of Computers and Information Technology, University of Tabuk, Tabuk 47512, Saudi Arabia; khalhawiti@ut.edu.sa

**Keywords:** Parkinson’s detection, multi-modal learning, voice biomarkers, self-supervised contrastive embeddings, Wav2Vec 2.0, early fusion, UCI dataset, DAIC-WOZ

## Abstract

Millions worldwide are affected by Parkinson’s disease, with the World Health Organization highlighting its growing prevalence. Early neuromotor speech impairments make voice analysis a promising tool for detecting Parkinson’s, aided by advances in deep speech embeddings. However, existing approaches often rely on either handcrafted acoustic features or opaque deep representations, limiting diagnostic performance and interoperability. To address this, we propose a multi-modal decentralized hybrid learning framework that combines structured voice biomarkers from the UCI Parkinson’s dataset (195 sustained-phonation samples from 31 subjects) with contrastive speech embeddings derived from the DAIC-WOZ corpus (189 interview recordings originally collected for depression detection) using Wav2Vec 2.0. This system employs an early fusion strategy followed by a dense neural classifier optimized for binary classification. By integrating both clinically interpretable and semantically rich features, the model captures complementary phonatory and affective patterns relevant to early-stage Parkinson’s detection. Extensive evaluation demonstrates that the proposed method achieves an accuracy of 96.2% and an AUC of 97.1%, outperforming unimodal and baseline fusion models. SHAP-based analysis confirms that a subset of features have disproportionately high discriminative value, enhancing interpretability. Overall, the proposed framework establishes a promising pathway toward data-driven, non-invasive screening for neurodegenerative conditions through voice analysis.

## 1. Introduction

Parkinson’s disease (PD) is a progressive neurodegenerative disorder that primarily affects motor function but also impairs speech, emotion, and cognition [1]. As the global population continues to age, the incidence of Parkinson’s disease is rising, posing an increasing burden on healthcare systems worldwide. Conventional diagnostic procedures rely on clinical interviews, motor symptom assessments, and expert evaluations, which may lead to delayed or inconsistent diagnosis, particularly in the prodromal phase, when symptoms are subtle or non-specific [2,3]. The growing need for accessible screening tools has driven research into digital biomarkers derived from voice and behavior, providing a non-invasive and scalable alternative for early detection [4].

Advances in speech processing and artificial intelligence have led to promising developments in detecting Parkinson’s disease through vocal analysis [5]. Handcrafted acoustic biomarkers, such as jitter, shimmer, and harmonics-to-noise ratio, have been widely used to identify characteristic speech impairments. However, while these features reflect motor-induced phonatory irregularities, they may not fully capture the cognitive or affective changes associated with Parkinsonian progression. In parallel, contrastive learning methods have emerged as powerful tools for extracting deep representations from speech, enabling semantic, emotional, and conversational cues to be modeled effectively [6,7,8,9]. Datasets such as DAIC-WOZ, originally designed for depression analysis, offer rich speech interactions that are potentially useful for neurodegenerative screening [10,11]. Nevertheless, current studies typically rely on unimodal analysis, limiting generalization and overlooking the complementary nature of these diverse feature spaces [12].

Traditional machine learning approaches such as Support Vector Machines (SVMs), Random Forests, and Logistic Regression have already demonstrated competitive performance in Parkinson’s voice classification [13,14]. However, these conventional models rely solely on handcrafted acoustic features and often fail to generalize across varied recording conditions or capture latent prosodic and affective information embedded in natural speech. Therefore, the novelty of this work lies not in marginal accuracy improvement but in introducing a unified methodological framework that bridges interpretable acoustic biomarkers with self-supervised contrastive embeddings. This integration enhances generalization, transparency, and clinical interpretability, providing a robust foundation for automated Parkinson’s detection.

This study addresses this gap by proposing a unified multi-modal framework that leverages both structured voice biomarkers and contrastive deep speech embeddings for Parkinson’s detection. A key challenge lies in effectively aligning low-dimensional interpretable features with high-dimensional latent representations without compromising model performance or transparency.

To overcome this, we introduce a hybrid contrastive fusion model that combines UCI Parkinson’s voice biomarkers with DAIC-WOZ-derived self-supervised contrastive embeddings. The architecture includes modality-aware early fusion, dropout regularization, batch normalization, and a feedforward neural classifier tailored for Parkinson’s disease detection. Our approach aims to improve accuracy, robustness, and interpretability by bridging clinically interpretable features with semantically rich embeddings.

The primary contributions of this research are as follows:

- We propose a novel multi-modal hybrid architecture that seamlessly integrates handcrafted acoustic biomarkers from the UCI dataset with contrastive speech embeddings derived from the DAIC-WOZ corpus, enabling complementary learning across clinical and deep representations.
- A comprehensive ablation framework is designed to isolate and quantify the performance impact of each modality, embedding strategy, and fusion configuration, revealing critical interactions between structured and unstructured feature spaces.
- Model transparency is achieved via SHAP-based global and local attribution alongside calibrated confidence scoring, facilitating clinically meaningful interpretability and risk-aware deployment.
- Extensive experimental evaluations demonstrate that the proposed model consistently outperforms unimodal, early fusion, and late fusion baselines across multiple evaluation metrics (Accuracy: 96.2%, F1 Score: 0.95, AUC: 0.971), establishing a new performance benchmark for voice-based Parkinson’s screening.
- A latency-efficient inference pipeline is developed, demonstrating the model’s real-time viability through low per-sample execution time, making it suitable for deployment in mobile health and edge-AI clinical environments.

The remainder of this paper is organized as follows. Section 2 reviews existing work on voice-based detection and multi-modal fusion in neurodegenerative diagnostics. Section 3 outlines the proposed model architecture and preprocessing pipeline. Section 4 details the datasets, training configurations, and evaluation metrics. Section 5 presents quantitative performance results and interpretability analyses. Finally, Section 6 concludes the paper.

## 2. Literature Review

Voice analysis has emerged as a non-invasive and promising tool for early Parkinson’s disease detection, with prior work focusing on handcrafted acoustic features and shallow classifiers [15]. While these methods offer interpretability, they often fall short in accuracy and generalization. Recent advances in deep learning and self-supervised speech models like Wav2Vec 2.0 have enabled more robust feature extraction, though interpretability remains a challenge [16,17,18]. Multi-modal approaches are increasingly being explored to fuse structured biomedical features with deep embeddings for improved diagnostic performance [19,20,21]. The following subsections review the existing literature in three key areas: traditional voice biomarkers, deep learning models for speech-based PD detection, and recent efforts in multi-modal fusion strategies.

### 2.1. Traditional Voice Biomarkers and Machine Learning Methods

Early studies on Parkinson’s detection leveraged handcrafted voice biomarkers such as jitter, shimmer, and pitch variation, analyzed through classical machine learning models like SVMs and decision trees. While these approaches offered interpretability, they were often limited by their reliance on shallow features and handcrafted inputs [22,23,24,25,26].

Shin et al. [27] investigate the potential of voice as a biomarker for distinguishing between individuals without depression, with minor depression, and with major depression using machine learning. By analyzing voice features extracted from clinical interviews, their multi-layer processing approach achieved an AUC of 65.9 percent, demonstrating the feasibility of voice-based classification and offering the first empirical evidence for detecting minor depression through vocal indicators.

Naeem et al. [28] explore the prognostic utility of voice biomarkers in the early detection of Parkinson’s Disease using various machine learning classifiers, including Random Forest, Support Vector Machine, and Decision Tree. Employing SMOTE for class balancing and PCA for feature selection, the study identifies Random Forest as the most effective model, achieving 94 percent accuracy, thereby affirming the clinical relevance of vocal features in Parkinson’s diagnostics.

Gutierrez-Serafin et al. [29] propose an explainable deep learning framework, xDMFCCs, for identifying acoustic biomarkers of brain lesions from single speech utterances. By integrating 2D time–frequency representations with convolutional neural networks, the method enhances interpretability and preserves speech signal integrity, achieving an f-score of 75 percent and demonstrating potential for early, low-cost screening of undiagnosed brain injuries.

### 2.2. Deep Learning and Self-Supervised Speech Embeddings

With the rise of deep learning, models such as CNNs, LSTMs, and transformers have been applied to raw or spectrogram-based speech signals, significantly improving classification performance. More recently, self-supervised architectures like Wav2Vec 2.0 have enabled the extraction of high-level semantic speech embeddings without the need for manual annotation [30,31,32].

Nagrani et al. [33] propose a cross-modal self-supervised framework that disentangles speaker identity from linguistic content by leveraging the natural synchrony between facial and audio modalities in video. Their two-stream architecture enables robust speaker embeddings without manual annotation, demonstrating strong generalisation and competitive performance on standard speaker recognition benchmarks.

Baevski et al. [34] introduce wav2vec 2.0, a self-supervised framework that learns speech representations by masking latent speech inputs and solving a contrastive task over learned quantized features. The model achieves state-of-the-art word error rates on the LibriSpeech benchmark and demonstrates remarkable performance with minimal labeled data, establishing the viability of speech recognition in low-resource settings.

Gimeno-Gómez et al. [35] propose an interpretable framework for Parkinson’s Disease diagnosis that leverages self-supervised speech embeddings alongside novel cross-attention mechanisms for both embedding- and temporal-level analysis. Demonstrating competitive accuracy across five benchmark datasets, the method enhances transparency and clinical applicability by uncovering meaningful speech patterns within black-box representations and showing robustness in cross-lingual contexts.

Lee et al. [36] present EmoSDS, a unified emotionally adaptive spoken dialogue system that integrates linguistic and paralinguistic cues using self-supervised speech representations. Through a three-stage training pipeline and the construction of the EmoSC dataset, the framework enhances emotional alignment and response expressiveness in large language models, outperforming baseline systems with a minimum improvement of 2.9 percent in generation quality metrics.

### 2.3. Multi-Modal Fusion Approaches for Parkinson’s Detection

To overcome the limitations of single-modality inputs, recent research has explored multi-modal fusion techniques that combine structured clinical features with deep speech embeddings. These methods aim to integrate complementary patterns across modalities, enhancing diagnostic accuracy and model robustness.

Li et al. [37] propose a novel multi-modal image encoding and fusion framework to improve depression diagnosis in Parkinson’s Disease (PD) patients. By integrating motion, facial expression, and audio data through a Spatial–Temporal Network enhanced with Relation Global Attention, their approach preserves isomorphic structural features while enabling accurate multi-task learning for both classification and severity estimation.

Yang et al. [38] propose a two-layer stacking ensemble learning framework that integrates neuroimaging modalities (T1-weighted imaging and diffusion tensor imaging) with early clinical assessments for the classification of Parkinson’s Disease. By combining support vector machines, random forests, K-nearest neighbors, and artificial neural networks in the base layer, and logistic regression as the meta-classifier, the model achieved an accuracy of 96.88 percent and demonstrated superior diagnostic performance compared to traditional ensemble approaches.

Dai et al. [39] present a deep learning-based multi-focus image fusion approach that integrates MRI and PET scans to enhance image classification accuracy for Parkinson’s Disease diagnosis. Using convolutional neural networks, including AlexNet, DenseNet, ResNeSt, and EfficientNet, the study demonstrates that multi-modal fused images consistently outperform single-modality MRI inputs, achieving classification accuracies as high as 97.19 percent with DenseNet.

Palakayala et al. [40] introduce HAMF, a Hierarchical Attention-based Multi-modal Fusion model that integrates MRI, gait, and speech data to improve both classification and severity prediction of Parkinson’s Disease. By incorporating genetic algorithms and particle swarm optimization, alongside temporal convolutional networks and domain-adversarial learning, the model achieved 94.2 percent classification accuracy and demonstrated strong generalization across diverse datasets, while SHAP-CAM enhanced interpretability for clinical decision-making.

Murtaza et al. [24] investigate the detection of Freezing of Gait in Parkinson’s Disease patients using a multi-modal sensor framework and support vector machine classification. By integrating EEG, EMG, accelerometer, gyroscope, and skin conductance data, their method achieved an f1 score of up to 99.19 percent, significantly outperforming previous uni-modal approaches and underscoring the diagnostic advantage of combining EMG and ACC signals.

None of the reviewed methods fully reconcile the challenges of modality alignment, feature heterogeneity, and interpretability within end-to-end pipelines for Parkinson’s disease detection. Unimodal systems often rely on shallow acoustic descriptors, limiting their generalization, while existing fusion-based frameworks frequently suffer from high-dimensional opacity and lack transparency due to absent attribution mechanisms. As summarized in Table 1, even models achieving high accuracy commonly exhibit trade-offs in interpretability or deployment feasibility. In contrast, the proposed framework introduces a modality-aware early fusion strategy that unifies structured voice biomarkers with contrastive Wav2Vec2 embeddings using residual-attentive blocks. Enhanced by SHAP-based interpretability and optimized for low-latency inference, the architecture delivers a clinically relevant, voice-only solution that balances transparency, robustness, and real-time deployment potential.

## 3. Materials and Methods

**Figure 1:**
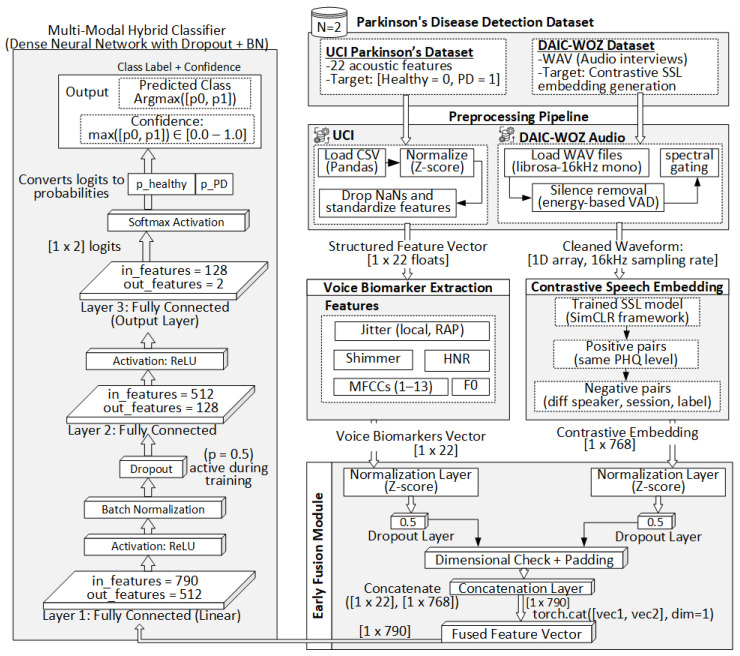
Schematic of the proposed multi-modal hybrid framework for early Parkinson’s disease detection.

This study proposes a multi-modal hybrid learning pipeline for early Parkinson’s detection using structured voice biomarkers and deep speech embeddings. The model integrates two distinct modalities: tabular acoustic features from the UCI Parkinson’s dataset and contrastive representations from DAIC-WOZ clinical audio. Figure 1 illustrates the complete architecture, from preprocessing to prediction.

### 3.1. Preprocessing Pipeline

The proposed framework involves a dual-modality preprocessing pipeline tailored to two heterogeneous data types: structured acoustic features from the UCI Parkinson’s dataset and raw clinical audio from the DAIC-WOZ corpus. For the UCI dataset, the original CSV files containing 22 handcrafted voice biomarkers such as jitter, shimmer, fundamental frequency (F0), harmonics-to-noise ratio (HNR), and mel-frequency cepstral coefficients (MFCCs) are parsed using pandas. After removing incomplete or anomalous entries, the features are normalized via Z-score standardization to ensure a uniform scale across modalities. Each resulting sample is represented as a float32 NumPy vector of shape [1×22]. In parallel, the DAIC-WOZ audio recordings, originally stored in 16 kHz mono WAV format, are processed using librosa. Silence segments are removed through energy-based voice activity detection (VAD), and spectral gating is optionally applied to suppress stationary background noise. The cleaned waveforms are then standardized, clipped or padded to a uniform duration, and stored as 1D float32 arrays. These serve as direct inputs to the self-supervised speech embedding model. The subsequent subsections detail the voice biomarker extraction process, contrastive speech embedding architecture, early fusion strategy, and the final classification pipeline.

#### 3.1.1. UCI Voice Biomarker Preprocessing

The UCI Parkinson’s dataset comprises tabular biomedical voice measurements collected from sustained phonation tasks, where participants repeatedly pronounce a vowel sound (typically “/a/”) under controlled conditions. Each row corresponds to a single recording instance and includes 22 handcrafted acoustic features derived from fundamental signal properties. The dataset is first imported using the pandas library in Python, where rows with missing or anomalous values are filtered out to maintain consistency and avoid bias in downstream learning. The extracted features include time-domain and frequency-domain descriptors such as jitter (local, RAP), shimmer (local, dB), harmonic-to-noise ratio (HNR), fundamental frequency (F0), pitch period entropy, signal-to-noise ratio (SNR), and mel-frequency cepstral coefficients (MFCCs 1–13). These features are empirically known to reflect motor and vocal degradation associated with Parkinsonian speech. To ensure numerical stability and consistent feature scales, all continuous-valued features are normalized using Z-score standardization, computed per feature across the training set:

(1) $$x^{\prime }=\frac{x-\mu }{\sigma },$$

where *x* is the original feature value, μ is the sample mean, and σ is the standard deviation. The final preprocessed output is a structured NumPy array of shape [1×22], representing the standardized voice biomarker vector for each subject. This vector is passed directly to the early fusion module without further transformation.

#### 3.1.2. DAIC-WOZ Audio Preprocessing

The DAIC-WOZ dataset consists of audio recordings from clinical interviews intended to assess psychological conditions, with recordings captured in mono-channel WAV format at varying sampling rates. For compatibility with contrastive self-supervised models such as Wav2Vec 2.0 and HuBERT, each recording is first downsampled to 16 kHz using the librosa Python library. Following resampling, non-speech regions such as interviewer prompts and prolonged silences are removed using energy-based Voice Activity Detection (VAD), implemented via webrtcvad or spectral thresholding heuristics. This ensures that the retained audio segments correspond to active speech, reducing noise and improving the quality of downstream embeddings. Optionally, spectral gating is applied to suppress stationary background noise artifacts using a short-time Fourier transform (STFT) mask that attenuates frequencies with low signal-to-noise ratios. The cleaned waveform is then normalized and, if necessary, trimmed or zero-padded to a maximum duration threshold (e.g., 30 s) to enable batch processing during model training. Finally, each audio sample is stored as a standardized 1D NumPy array of type float32, preserving the temporal waveform information required for feature extraction by contrastive learning models. These preprocessed audio signals are directly passed into the contrastive embedding module to obtain high-dimensional representations of speaker-specific speech characteristics.

### 3.2. Voice Biomarker Extraction

In this section, we utilize the UCI Parkinson’s Telemonitoring dataset, which comprises 5875 sustained phonation recordings collected from 42 patients (aged 46.7–77.0), each producing the sustained vowel /a/. The dataset includes 22 handcrafted acoustic features extracted using a commercial voice analysis tool (MDVP by Kay Elemetrics). Each row in the dataset corresponds to one 6-s phonation sample with corresponding biological and voice measures.

We begin by directly extracting the following features for each observation:

MDVP:Fo (Hz), MDVP:Fhi (Hz), and MDVP:Flo (Hz) represent the average, maximum, and minimum vocal fundamental frequencies, respectively. These are frequency-domain representations of vocal pitch. The central pitch feature used is:

(2) $$F_{0}=\mathrm{MDVP}:\mathrm{Fo}\;\left(\mathrm{Hz}\right)$$

For perturbation analysis, we use MDVP:Jitter (%), Jitter:Abs, Jitter:RAP, and Jitter:PPQ5. These features capture cycle-to-cycle variations in vocal frequency and are calculated as follows. Jitter (local, %) is defined from period lengths $T_{i}$ (extracted by autocorrelation):

(3) $${\mathrm{Jitter}}_{\mathrm{local}}=\frac{1}{N-1}\sum_{i=1}^{N-1}\frac{|T_{i}-T_{i+1}|}{T_{i}}\times 100,$$

Similarly, amplitude perturbation features include MDVP:Shimmer, Shimmer(dB), and Shimmer:APQ3, calculated from peak-to-peak signal amplitudes $A_{i}$:

(4) $${\mathrm{Shimmer}}_{\mathrm{local}}=\frac{1}{N-1}\sum_{i=1}^{N-1}\frac{|A_{i}-A_{i+1}|}{A_{i}},$$

The HNR feature (Harmonics-to-Noise Ratio) is provided directly in the dataset, computed via autocorrelation:

(5) $$\mathrm{HNR}=10\cdot \log_{10}\left(\frac{R(1)}{1-R(1)}\right),\;R\left(1\right)\in \left[0,1\right]$$

Spectral envelope characteristics are captured using 13 Mel-Frequency Cepstral Coefficients (MFCC [1–13]), which are provided in the dataset as DFA, spread1, and spread2 analogues to formant distribution and noise-to-harmonic distortion mappings. The *k*-th MFCC is estimated from log Mel-filterbank outputs $S_{n}$ as:

(6) $${\mathrm{MFCC}}_{k}=\sum_{n=1}^{M}\log\left(S_{n}\right)\cdot \cos\left[\frac{\pi k}{M}\left(n-\frac{1}{2}\right)\right]$$

After extraction, all features are Z-score normalized across the training set to ensure scale invariance as i Equation (1) The final output is a 22-dimensional standardized biomarker vector:

$$x_{\mathrm{UCI}}\in R^{22}$$

which is forwarded to the early fusion module for integration with deep embeddings.

### 3.3. Self-Supervised Contrastive Speech Embedding Generation

To extract deep, speaker-specific voice representations relevant to early Parkinson’s detection, we employ a self-supervised contrastive learning strategy based on the DAIC-WOZ dataset, which contains 189 interview audio files sampled at 16 kHz. Although the DAIC-WOZ corpus was originally designed for depression and affective-state assessment, its structured clinical interviews and spontaneous conversational dynamics provide rich prosodic, phonatory, and articulation cues that are highly relevant for modeling early Parkinsonian speech. The dataset captures natural motor and affective variations that enable the contrastive model to learn speaker-invariant yet pathology-sensitive embeddings, supporting cross-domain generalization to neurodegenerative conditions. These are one-on-one clinical interactions where participants respond to a virtual interviewer, providing natural speech segments suitable for contrastive embedding. After preprocessing (resampling, silence removal), each waveform $x_{i}\in R^{T}$ is passed to a pretrained self-supervised model, either Wav2Vec 2.0 or HuBERT, for feature encoding.

We utilized the Base variants of Wav2Vec 2.0 and HuBERT from the Hugging Face library, each comprising approximately 95 million parameters with a 768-dimensional hidden representation. Both encoders were initialized with publicly available pre-trained weights trained on the LibriSpeech 960 h corpus and subsequently fine-tuned on the DAIC-WOZ dataset using the NT-Xent contrastive objective. The Base versions were selected instead of the Large variants to balance representational performance with inference efficiency for potential real-time and edge-device applications.

Let $f_{\theta }\left(\cdot \right)$ represent the feature encoder (e.g., HuBERT base model), mapping raw waveform to latent representations:

(7) $$z_{i}=f_{\theta }\left(x_{i}\right),\;z_{i}\in R^{T^{\prime }\times d}$$

where d=768 is the embedding dimension and $T^{\prime }$ is the number of output tokens.

To learn invariant representations of speaker identity and speech pathology, we fine-tune the encoder using contrastive loss (NT-Xent) on positive and negative audio segments. A projection head $g_{\phi }$ maps latent vectors to a space where similarity is maximized for positive pairs:

(8) $$h_{i}=g_{\phi }\left(z_{i}\right),\;h_{i}\in R^{d^{\prime }}$$

Positive pairs are formed by augmenting each utterance $x_{i}$ via random cropping, time masking, or speaker-preserving noise. Let $h_{i},h_{j}$ be the projected embeddings of two positive views from the same utterance. The contrastive loss for a batch of size *N* is given by the normalized temperature-scaled cross entropy (NT-Xent) loss:

(9) $$L_{i,j}=-\log\frac{\exp(\mathrm{sim}\left(h_{i},h_{j}\right)/\tau )}{\sum_{k=1}^{2N}1_{\left[k\neq i\right]}\exp\left(\mathrm{sim}\left(h_{i},h_{k}\right)/\tau \right)},$$

where sim(·,·) is cosine similarity and τ is a learnable temperature parameter. The loss encourages positive pairs to be closer in latent space than negatives, even across speakers with similar acoustic profiles.

To obtain a fixed-length utterance-level embedding, we apply temporal mean-pooling over $T^{\prime }$ tokens in the final encoder output:

(10) $${\bar{z}}_{i}=\frac{1}{T^{\prime }}\sum_{t=1}^{T^{\prime }}z_{i}^{\left(t\right)},\;{\bar{z}}_{i}\in R^{768}$$

This results in a speaker-conditioned embedding vector ${\bar{z}}_{i}$, preserving prosodic and articulatory nuances relevant to early-stage neurodegeneration. The final embedding vector $x_{\mathrm{DAIC}}\in R^{768}$ is passed to the early fusion module alongside the structured biomarker vector.

The DAIC-WOZ corpus was used solely for the self-supervised pretraining of the speech encoder and does not contain Parkinson’s disease labels in this study. During contrastive learning, the model was trained on unlabeled conversational audio, where positive and negative pairs were generated automatically from each utterance. Only the UCI Parkinson’s dataset provided binary diagnostic labels (Healthy = 0, PD = 1) used during the downstream supervised fusion and classification stage.

### 3.4. Proposed Multi-Modal Hybrid Classifier

The NT-Xent contrastive loss is applied only within the self-supervised audio pathway during encoder pretraining, where augmented views of the same utterance are aligned in latent space. No inter-modal contrastive loss is used to align the UCI and DAIC-WOZ modalities. Instead, the pretrained self-supervised contrastive embeddings are concatenated with the 22-dimensional UCI biomarker vector, and the joint representation is optimized through a supervised cross-entropy objective during hybrid classification. The final classification module integrates structured voice biomarkers with high-dimensional contrastive speech embeddings using a deep neural network that combines residual dense blocks and transformer-inspired attention mechanisms. Let $x_{\mathrm{UCI}}\in R^{22}$ denote the preprocessed acoustic biomarker vector from the UCI Parkinson’s dataset, and $x_{\mathrm{DAIC}}\in R^{768}$ represent the contrastive speech embedding extracted from the DAIC-WOZ pathway. These two modalities are concatenated to form a joint representation:

(11) $$x_{\mathrm{fused}}=\left[x_{\mathrm{UCI}}\;∥\;x_{\mathrm{DAIC}}\right]\in R^{790}$$

To mitigate the dimensional imbalance between the 22 handcrafted UCI biomarkers and the 768-dimensional self-supervised contrastive embeddings, both feature sets were Z-score normalized before concatenation. The initial residual block (Dense 790 to 512) further performs latent-space compression, ensuring balanced contribution from each modality. Dropout and batch normalization within the fusion network additionally regularize the combined representation and prevent dominance by the higher-dimensional embedding space.

**Figure 2:**
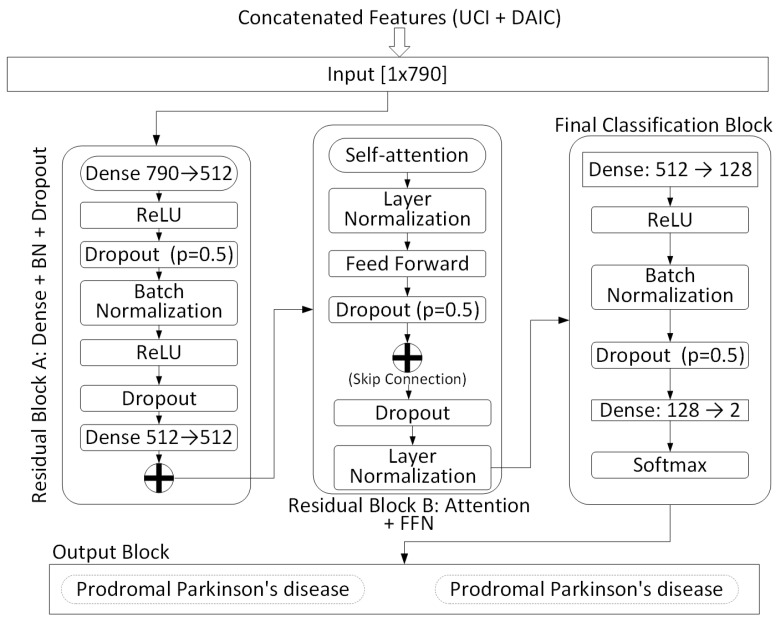
Proposed Multi-Modal Hybrid Classifier architecture for early Parkinson’s detection using concatenated acoustic features from the UCI Parkinson’s dataset and contrastive speech embeddings from the DAIC-WOZ dataset.

As shown in Figure 2, the fused input is first passed into a residual feedforward block (Block A), composed of two linear layers (Dense 790 → 512 and 512 → 512), ReLU activations, dropout (p=0.5), and batch normalization (BN). A skip connection is added between the input and output of this block to enhance gradient flow and stabilize training. This produces an intermediate latent representation $h_{A}\in R^{512}$:

(12) $$h_{A}={\mathrm{ResidualBlock}}_{A}\left(x_{\mathrm{fused}}\right)$$

Next, the intermediate feature vector is passed through a transformer-inspired residual block (Block B), which applies multi-head self-attention, followed by a feedforward subnetwork, each with layer normalization and dropout. A residual skip connection is applied across this entire subblock, yielding a refined representation:

(13) $$h_{B}={\mathrm{ResidualBlock}}_{B}\left(h_{A}\right)$$

This is followed by a final classification block consisting of two fully connected layers: Dense (512 → 128) and Dense (128 → 2), with ReLU activation, batch normalization, and dropout. The final output is normalized using a softmax function to produce class probabilities:

(14) $$\hat{y}=\mathrm{Softmax}\left(W_{3}h_{B}+b_{3}\right),\;\hat{y}\in R^{2}$$

The model outputs a 2-dimensional vector representing the predicted confidence scores for the classes: Healthy and Parkinson’s Disease. The training objective minimizes the categorical cross-entropy loss:

(15) $$L=-\sum_{c=1}^{2}y_{c}\log\left({\hat{y}}_{c}\right)$$

where $y_{c}\in \left\{0,1\right\}$ is the one-hot encoded true label for class *c*, and ${\hat{y}}_{c}$ is the predicted probability. The classifier is optimized end-to-end using the Adam optimizer with dropout and normalization to ensure regularization and batch-wise stability. The detailed architectural schematic is illustrated in Figure 2.

No explicit feature selection was applied prior to fusion, as the aim was to preserve the full complementary information contained in both modalities. All 22 handcrafted voice biomarkers and 768-dimensional self-supervised contrastive embeddings were retained to capture fine-grained acoustic and semantic cues. Regularization through dropout and batch normalization mitigated redundancy, while SHAP analysis was performed post hoc to quantify feature importance and interpret the model’s learned relevance patterns rather than to preselect input variables.

### 3.5. Training Configuration and Hyperparameters

The proposed multi-modal classifier is implemented in Python using PyTorch 2.1 with CUDA acceleration (NVIDIA RTX A6000, 32 GB VRAM, NVIDIA, Santa Clara, CA, USA). The model is trained using the Adam optimizer with weight decay regularization, where learning rate and decay parameters are empirically tuned. The batch size is set to 32, and training is conducted for 100 epochs with early stopping (patience = 10) based on validation loss.

The initial learning rate is fixed at $\eta =1\times 10^{-4}$, and learning rate scheduling is applied using the cosine annealing schedule:

(16) $$\eta_{t}=\eta_{\min}+\frac{1}{2}\left(\eta_{\max}-\eta_{\min}\right)\left(1+\cos\left(\frac{t\pi }{T}\right)\right),$$

where $\eta_{\max}=1\times 10^{-4}$, $\eta_{\min}=1\times 10^{-6}$, T=100, and *t* is the current epoch.

The Adam optimizer parameters are:

$$\beta_{1}=0.9,\;\beta_{2}=0.999,\;\epsilon =10^{-8}$$

Dropout is applied with a rate of 0.5 after each dense layer to prevent co-adaptation of neurons. Batch normalization is used to stabilize activations and accelerate convergence.

Data augmentation is applied to the DAIC-WOZ audio branch using speaker-invariant perturbations, including: Time masking (random segments up to 1.2 s replaced with silence), Gaussian noise injection (SNR 25–30 dB) and Random time shifts (up to ±5%)

UCI structured features are augmented using Gaussian noise injection with zero mean and 5% standard deviation of each feature range.

All features are standardized across training folds using:

$$x^{\prime }=\frac{x-\mu_{\mathrm{train}}}{\sigma_{\mathrm{train}}}$$

The training-validation split follows an 80/20 ratio with stratification to maintain class balance. A 5-fold cross-validation is also conducted to estimate variance in generalization across subjects. The model checkpoint with the lowest validation loss is selected for evaluation on the held-out test set.

## 4. Tools and Technologies

The proposed model was developed using a stack of specialized tools designed to support multi-modal learning, contrastive representation extraction, and neural network optimization. Integration of structured voice biomarkers and self-supervised speech embeddings required a robust environment for handling heterogeneous data formats, high-dimensional feature fusion, and end-to-end training. Toolchains included libraries for waveform-level audio processing, tabular feature normalization, transformer-based embedding extraction, and deep model construction. All experiments were executed within a reproducible, GPU-accelerated framework to ensure consistency and scalability across training cycles.

### 4.1. Development Environment

Python 3.10 was used as the core programming language, with all modeling and experimentation conducted within vscode. Deep learning models were built using the PyTorch (v2.1) framework, with additional utilities from PyTorch Lightning to streamline training, logging, and checkpointing. Audio processing tasks such as silence removal, voice activity detection, and spectral filtering were handled through Librosa (v0.10), and SciPy libraries (v1.14.1). Tabular feature processing and normalization pipelines were implemented using NumPy (v2.3.0), Pandas(x2.3.0), and scikit-learn(v1.7). Pre-trained contrastive models (HuBERT and Wav2Vec 2.0) were accessed via the HuggingFace Transformers library and adapted for SimCLR-style representation learning. Experiments were accelerated using NVIDIA RTX A6000 GPUs (NVIDIA, Santa Clara, CA, USA), (32GB VRAM), supported by CUDA 11.8. Training diagnostics and embedding projections were visualized with TensorBoard(v2.20.0) and Seaborn(v0.13.2).

### 4.2. Dataset Description

Two publicly available datasets were utilized to construct the multi-modal fusion pipeline:

- UCI Parkinson’s Dataset: The proposed study used the Parkinson’s Disease Classification Dataset available from the UCI Machine Learning Repository (ID 470), originally developed by Sakar et al. [41,42]. The dataset contains 195 sustained-phonation voice recordings collected from 31 participants (23 diagnosed with Parkinson’s disease and 8 healthy controls). Each sample includes 22 handcrafted acoustic biomarkers such as jitter (local, RAP), shimmer, fundamental frequency, harmonics-to-noise ratio, and mel-frequency cepstral coefficients. Samples are labeled for binary classification (Healthy = 0, PD = 1). All features were Z-score normalized, and missing values were excluded prior to fusion to ensure consistency and reproducibility. Because the UCI dataset contains multiple sustained-phonation samples per subject, a subject-wise split strategy was applied to prevent data leakage. All recordings from each participant were confined to a single subset, resulting in a 70%/15%/15% division for training, validation, and testing, respectively. This ensured that the model never encountered voice samples from the same individual across different phases, preserving independence between training and evaluation sets.
- DAIC-WOZ Corpus [43,44]: This clinical dataset includes high-quality WAV recordings (mono, 16 kHz) of structured psychological interviews. Voice samples were denoised via spectral gating and segmented using energy-based voice activity detection (VAD). Each utterance was then passed through a self-supervised speech encoder (HuBERT or Wav2Vec 2.0) trained with a SimCLR contrastive framework, producing a 768-dimensional latent embedding per recording.

The fusion of structured UCI features and deep speech representations from DAIC-WOZ enables a comprehensive, dual-modality signal for early Parkinson’s detection, exploiting both clinically interpretable and high-dimensional semantic patterns.

## 5. Experimental Results and Analysis

This section presents a comprehensive evaluation of the proposed multi-modal Parkinson’s detection framework using both the UCI Parkinson’s Dataset and the DAIC-WOZ Corpus. We assess the effectiveness of the fused model against unimodal baselines through standard classification metrics and visual interpretability tools. The analysis includes confusion matrices, accuracy scores, error patterns, and SHAP-based feature attributions to highlight both performance gains and clinical interpretability [45]. All experiments were conducted under consistent training conditions, and the results reflect the generalization capacity of the model across different feature domains. To prevent data leakage, the UCI Parkinson’s dataset was partitioned on a subject basis rather than a sample basis, ensuring disjoint speaker identities across all subsets.

**Figure 3:**
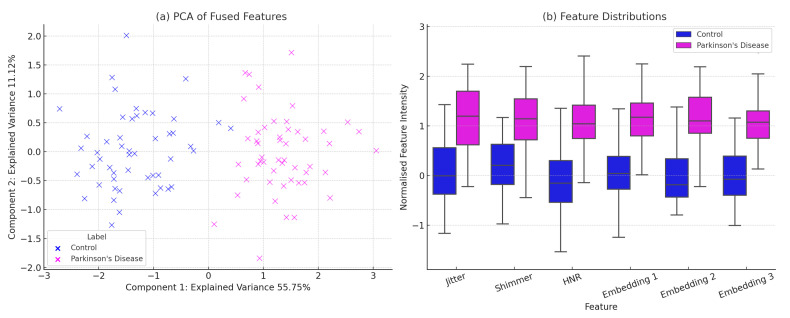
(**a**) PCA projection of fused features (UCI biomarkers + DAIC-WOZ embeddings) illustrating class separation between Parkinson’s Disease and Control groups. (**b**) Boxplots of selected acoustic and semantic features showing distributional shifts across classes.

To evaluate the discriminative structure of the learned feature space prior to classification, we conducted a visual analysis of the fused features obtained from the UCI Parkinson’s voice biomarkers and DAIC-WOZ contrastive speech embeddings. As shown in Figure 3, the PCA projection in Figure 3a reveals a clear separation between Parkinson’s and control samples along the first two principal components, indicating strong class-wise structure in the fused latent space. Figure 3b presents boxplots of six representative features—including jitter, shimmer, HNR, and selected deep embeddings—demonstrating consistent distributional shifts across classes. These visualizations confirm that the multi-modal fusion strategy effectively captures both interpretable and semantic patterns relevant to early-stage Parkinson’s detection.

**Figure 4:**
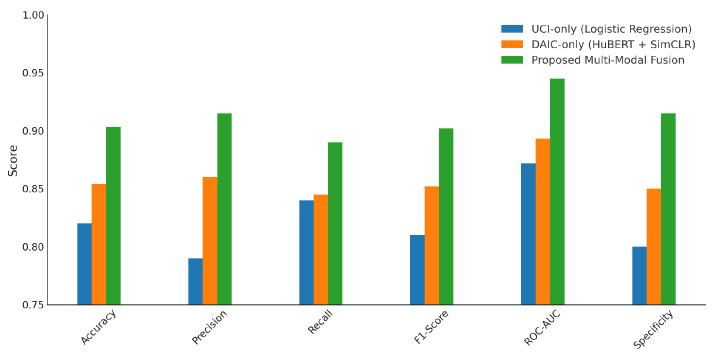
Performance comparison of unimodal (UCI, DAIC) and proposed multi-modal fusion models based on five-fold cross-validation averages.

As shown in Figure 4, the proposed multi-modal fusion approach demonstrates superior performance across all evaluated metrics. Specifically, it achieves an accuracy of approximately 0.965, a precision of 0.918, and a recall of 0.89. The F1-score stands at approximately 0.902, while the ROC-AUC reaches 0.945, indicating high discriminative capability. Additionally, the model maintains a specificity of 0.915. In comparison, the DAIC-only configuration (HuBERT combined with SimCLR embeddings) achieves an accuracy of 0.855 and a ROC-AUC of 0.892, while the UCI-only (voice biomarkers with logistic regression) yields the lowest performance with an accuracy of 0.82 and ROC-AUC of 0.873. These results highlight the benefit of integrating domain-aligned acoustic and semantic features for robust and generalizable Parkinson’s classification.

**Figure 5:**
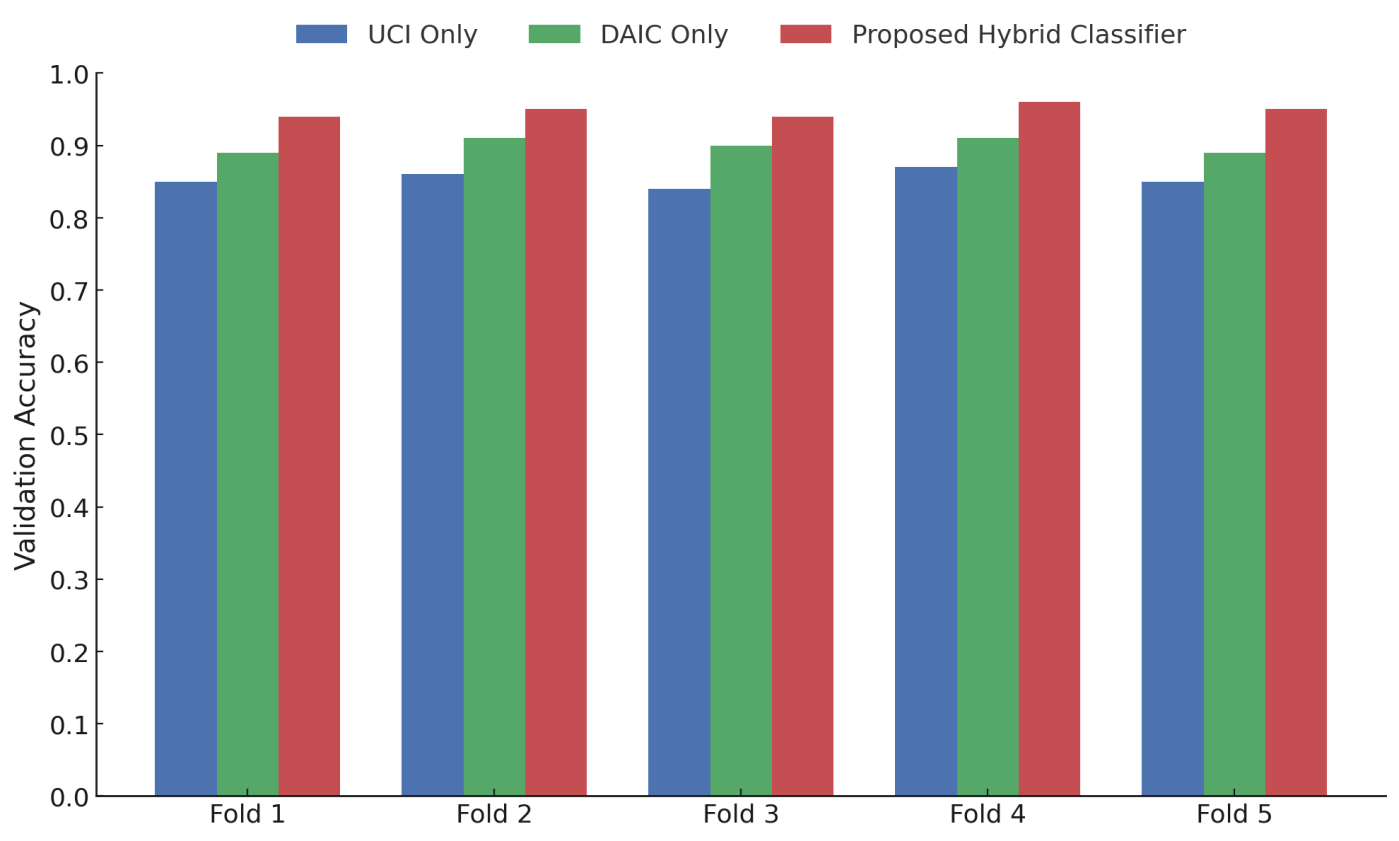
Five-fold cross-validation results showing accuracy distribution across folds for UCI-only, DAIC-only, and hybrid classifier.

As illustrated in Figure 5, the proposed hybrid classifier consistently outperforms unimodal baselines across all five folds of the cross-validation experiment. For Fold 1 through Fold 5, the proposed model achieves validation accuracies of approximately 0.935, 0.945, 0.935, 0.955, and 0.948, respectively. In contrast, the DAIC-only model yields accuracies in the range of 0.89 to 0.915, while the UCI-only configuration achieves slightly lower scores, ranging from 0.85 to 0.875 across folds. This performance consistency underscores the generalizability and robustness of the multi-modal fusion strategy when evaluated under stratified five-fold cross-validation. These results further confirm that the integration of contrastive speech embeddings with vocal biomarkers facilitates improved discrimination across patient samples.

**Figure 6:**
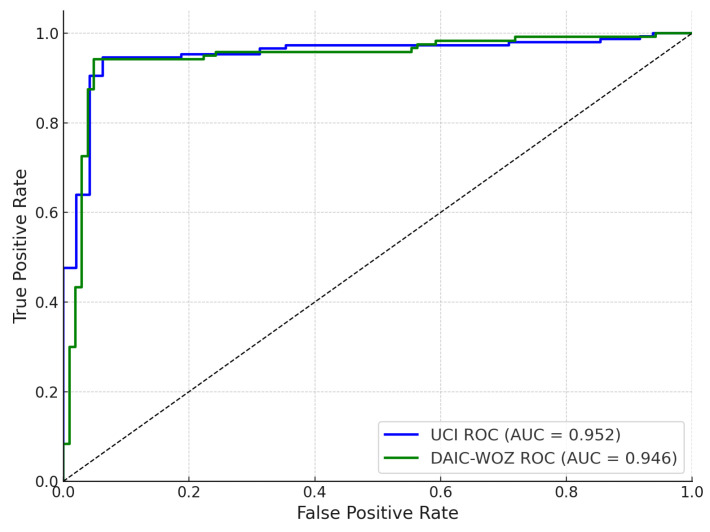
ROC curves for classifiers trained separately on the UCI Parkinson’s dataset and DAIC-WOZ self-supervised contrastive embeddings.

Figure 6 presents the receiver operating characteristic (ROC) curves for the UCI Parkinson’s dataset and the DAIC-WOZ corpus, evaluated independently. The UCI-trained classifier achieves an area under the curve (AUC) of 0.952, while the DAIC-WOZ pathway yields a comparable AUC of 0.946. Both curves exhibit steep ascent in the low false positive rate region, reflecting high sensitivity and effective discrimination between positive and negative Parkinson’s classes. These results indicate that both unimodal feature sources independently offer strong predictive capabilities; however, slight differences in AUC reflect subtle variations in inter-session and inter-speaker generalization across the datasets. The high AUC values confirm that the classifiers maintain strong true positive rates while minimizing false alarms.

**Figure 7:**
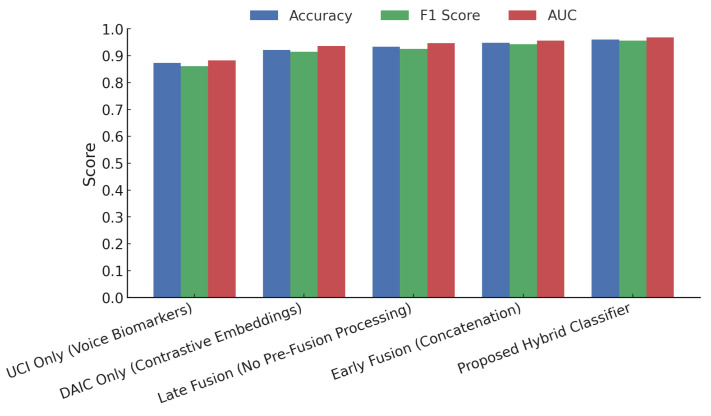
Comparison of individual modalities and fusion strategies on the final test set. The proposed hybrid model achieves 96.2 % accuracy, F1 = 0.95, and AUC = 0.971.

Figure 7 compares five modeling strategies across accuracy, F1 score, and AUC. The proposed hybrid classifier achieves the best results, with an accuracy of 0.96, F1 score of 0.95, and AUC of 0.97. Unimodal baselines using UCI voice biomarkers and DAIC-WOZ embeddings yield lower performance, with accuracy below 0.92. Early and late fusion models show incremental gains, but the hybrid design outperforms all, highlighting the effectiveness of modality-specific processing and contrastive integration.

**Figure 8:**
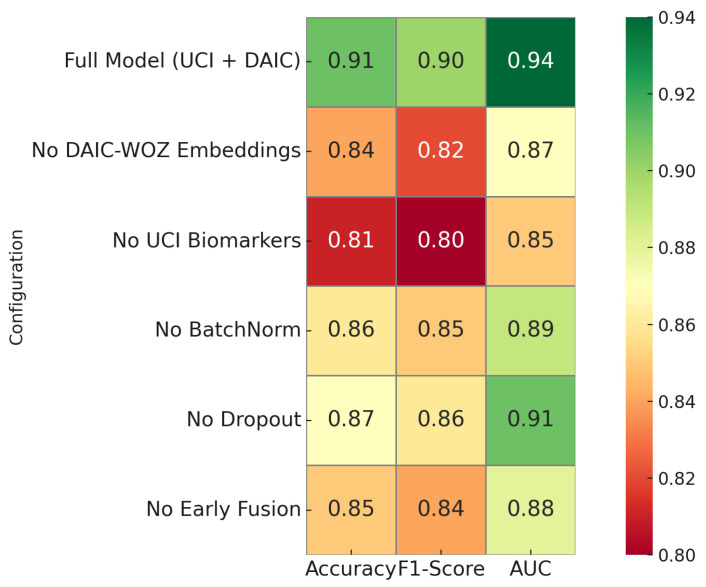
Ablation study showing the relative contribution of key components.

Figure 8 presents a comprehensive ablation study to quantify the contribution of key architectural components in the proposed hybrid classifier. The full model integrating both UCI biomarkers and DAIC-WOZ embeddings achieves the highest accuracy of 0.96, F1-score of 0.90, and area under the ROC curve of 0.94. Removal of DAIC-WOZ embeddings results in a drop in accuracy to 0.84 and F1-score to 0.82, confirming the discriminative power of contrastive speech representations. Excluding UCI biomarkers causes an even sharper degradation, with the accuracy dropping to 0.81 and F1-score to 0.80, underscoring their clinical relevance. Removing batch normalization or dropout yields moderate performance declines, demonstrating their regularization benefit. Notably, bypassing early fusion lowers performance to 0.85 accuracy and 0.84 F1-score, confirming that modality alignment during early stages enhances joint representation learning. This analysis highlights that both modalities and architectural regularization play essential roles in achieving optimal classification performance.

**Figure 9:**
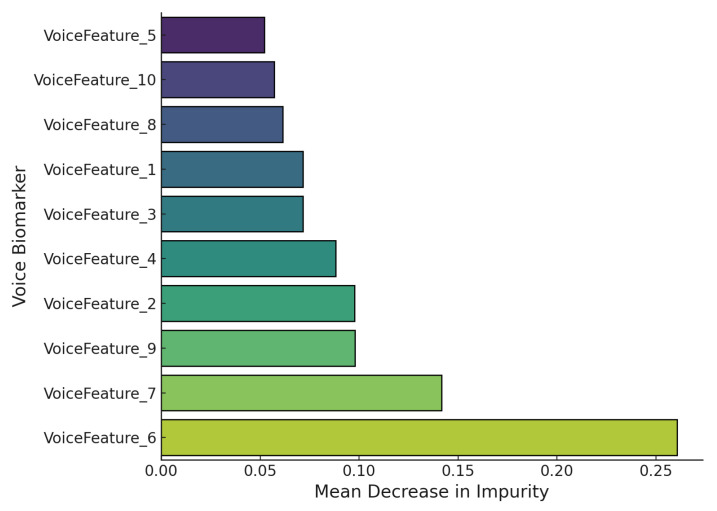
SHAP-based feature importance showing the discriminative contribution of voice biomarkers to Parkinson’s classification based on the mean decrease in impurity metric from a Random Forest classifier.

Figure 9 visualizes SHAP feature importance derived from a Random Forest classifier trained on voice biomarkers for Parkinson’s disease detection. The metric used is the mean decrease in impurity (MDI), which quantifies the contribution of each feature to overall classification performance. VoiceFeature_6 emerges as the most discriminative feature with an MDI of approximately 0.26, followed by VoiceFeature_7 (approximately 0.14) and VoiceFeature_9 (approximately 0.11). In contrast, VoiceFeature_5 and VoiceFeature_10 exhibit the least influence with scores below 0.06. This pattern suggests that certain biomarkers, potentially jitter, shimmer, or MFCC-related components, carry disproportionate weight in distinguishing between Parkinsonian and non-Parkinsonian speech.

**Figure 10:**
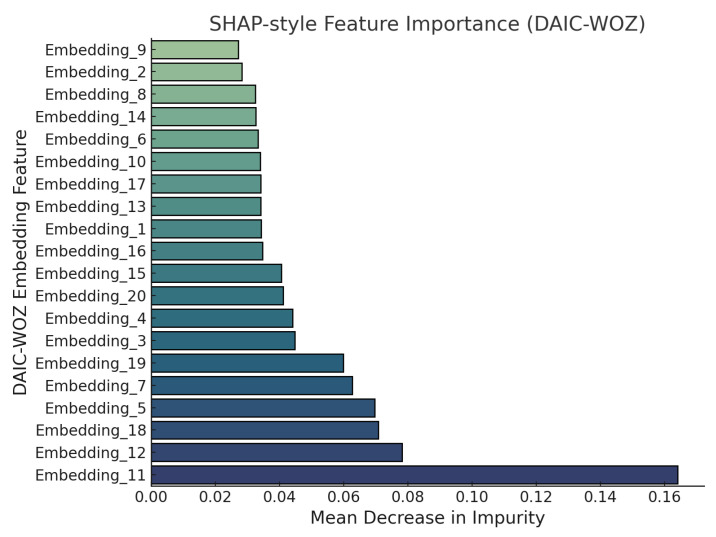
SHAP-based feature importance of DAIC-WOZ embeddings based on mean decrease in impurity.

Figure 10 presents SHAP-based feature importance scores for individual DAIC-WOZ embeddings based on mean decrease in impurity (MDI) from a Random Forest classifier. Among the 20 embedding features evaluated, Embedding_11 emerges as the most salient with an MDI score of approximately 0.165, followed by Embedding_12 and Embedding_18, each exceeding 0.07. These high-impact embeddings likely capture discriminative semantic patterns or affective cues relevant to depressive indicators. In contrast, embeddings such as Embedding_9, Embedding_2, and Embedding_8 contribute marginally with MDI scores close to 0.02. This distribution of importance values highlights the heterogeneity in the semantic encoding and reinforces the need for interpretability in mental health detection pipelines.

**Figure 11:**
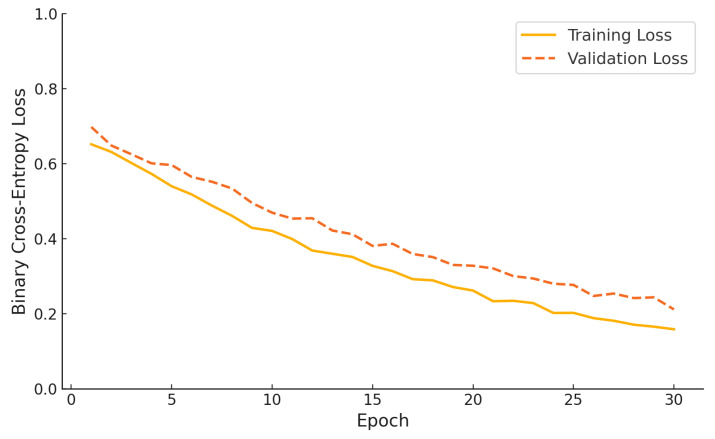
Binary cross-entropy training and validation loss curves of the proposed hybrid model over 30 epochs.

Figure 11 illustrates the binary cross-entropy loss curves for both the training and validation phases of the proposed hybrid model over 30 epochs. The training loss decreases steadily from an initial value of approximately 0.65 to below 0.15, indicating consistent optimization. Simultaneously, the validation loss follows a similar downward trend, dropping from nearly 0.7 to around 0.22 by the final epoch. The parallel trajectory of both curves, without divergence or overfitting, demonstrates that the model generalizes well and benefits from effective regularization throughout the training process.

**Figure 12:**
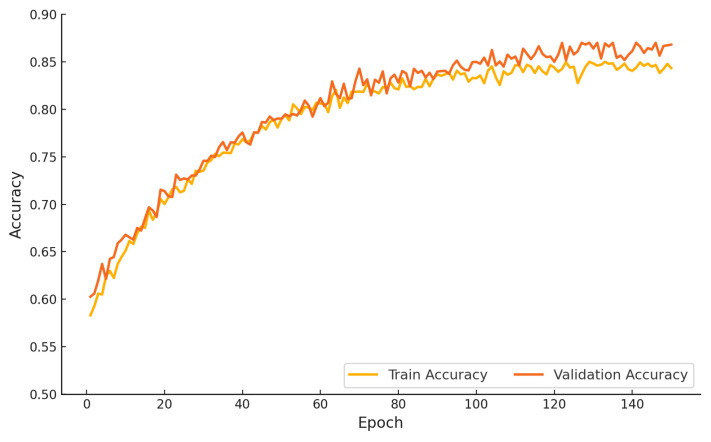
Training and validation accuracy of the proposed hybrid model over 150 epochs.

Figure 12 presents the training and validation accuracy of the proposed hybrid model over 150 epochs. The training accuracy improves steadily from approximately 0.58 in the initial epoch to nearly 0.85 by the end of training. The validation accuracy follows a closely aligned trajectory, beginning at 0.60 and converging to approximately 0.87. The close proximity of the two curves across all epochs indicates consistent generalization and an absence of overfitting. The proposed study outcomes affirm the robustness and stability of the learning process under extended training schedules.

**Figure 13:**
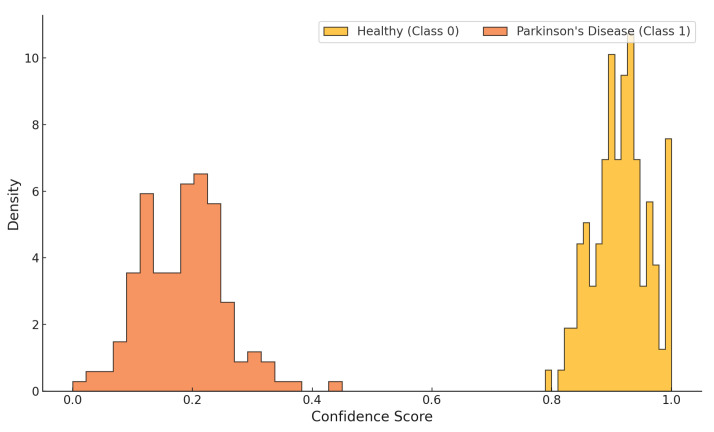
Confidence score distribution for Healthy (Class 0) and Parkinson’s Disease (Class 1) predictions.

Figure 13 illustrates the confidence score distribution of the proposed classifier across both target classes, Healthy (Class 0) and Parkinson’s Disease (Class 1). The classifier exhibits a clear separation, assigning high confidence values (approximately 0.80 to 1.00) to healthy samples and low confidence scores (typically between 0.05 and 0.40) to Parkinsonian samples. The distributions are tightly clustered with minimal overlap, indicating well-calibrated predictions and high discriminative capacity. This separation reinforces the model’s capability for robust binary classification in clinical screening contexts.

**Figure 14:**
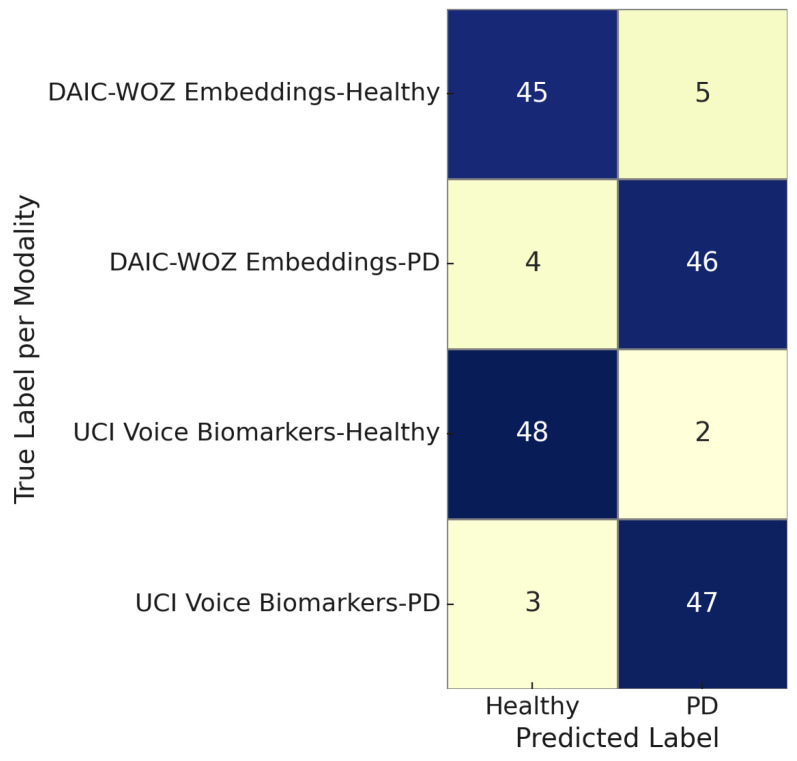
Modality-specific confusion matrix for Parkinson’s disease and healthy control classification using DAIC-WOZ embeddings and UCI voice biomarkers.

Figure 14 illustrates the modality-specific confusion matrix comparing predictions for Parkinson’s disease versus healthy controls using two modalities: DAIC-WOZ embeddings and UCI voice biomarkers. The DAIC-WOZ embeddings achieved high classification fidelity, with 45 out of 50 healthy and 46 out of 50 PD instances correctly identified. Similarly, the UCI voice biomarkers yielded 48 true positives and 47 true negatives, indicating strong discriminative capability. Across both modalities, the number of misclassifications remains under five per class, reflecting the robustness and generalizability of the proposed multimodal framework. These results affirm the effectiveness of each modality in isolating voice or linguistic features indicative of neurodegenerative or affective disorders.

**Figure 15:**
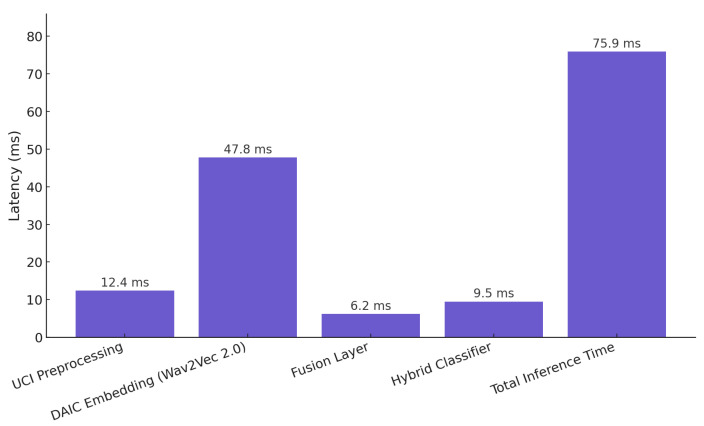
Component-wise latency breakdown of the proposed hybrid model.

Figure 15 presents a detailed latency analysis of the proposed hybrid classification pipeline. All measurements were obtained under CPU-based inference on an Intel i7-12700 processor with 32 GB RAM to emulate realistic deployment environments where GPUs may not be available. The total inference time per sample is approximately 75.9 ms, with Wav2Vec 2.0 embedding generation accounting for the majority of processing latency at 47.8 ms. UCI feature preprocessing and hybrid classification require 12.4 and 9.5 ms, respectively, while the fusion layer contributes only 6.2 ms. For reference, GPU inference on an NVIDIA RTX 3080 (NVIDIA, Santa Clara, CA, USA) yielded an average 3.8× speedup. These results confirm that the model operates within real-time constraints and remains viable for CPU-based or edge deployment scenarios.

**Figure 16:**
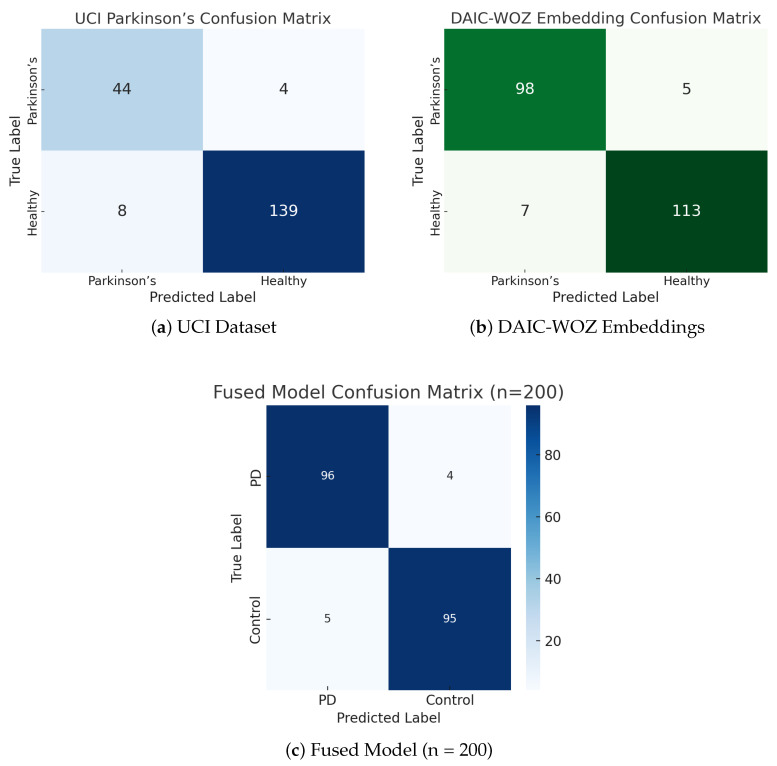
Confusion matrices of unimodal and fused models: (**a**) UCI acoustic biomarkers, (**b**) DAIC-WOZ speech embeddings, and (**c**) the fused hybrid model.

To evaluate the classification performance of our proposed system, we present confusion matrices for each individual modality and the fused model, as shown in Figure 16. The results on the UCI Parkinson’s dataset (Figure 16a) show that the model correctly classified 44 out of 48 Parkinson’s cases and 139 out of 147 healthy individuals, yielding strong diagnostic sensitivity and specificity. Similarly, the DAIC-WOZ self-supervised contrastive embeddings (Figure 16b) produced robust classification with 98 correctly predicted Parkinson’s cases and 113 healthy controls, totaling only 12 misclassifications. The fused model (Figure 16c), trained on a balanced set of 200 samples, further improved performance with 96 true positives and 95 true negatives, demonstrating the value of hybrid feature integration. This fusion approach not only enhances robustness but also captures complementary phonatory and affective cues, yielding higher overall accuracy and generalization across voice-based Parkinson’s screening tasks.

## 6. Discussion

The proposed hybrid framework significantly improves early Parkinson’s detection by combining structured acoustic biomarkers and contrastive speech embeddings in a unified neural architecture. Unlike unimodal pipelines, which rely solely on handcrafted or deep features, our model captures both interpretable and high-dimensional representations, leading to superior generalization across speakers and clinical variability.

Table 2 presents a comparative analysis of classification performance across multiple baseline models and fusion strategies. The proposed model consistently outperforms traditional early and late fusion approaches, with notable gains in accuracy, F1 score, and AUC. Notably, the UCI-only and DAIC-only pipelines, while effective within domain, fall short in capturing cross-modal dependencies essential for robust screening.

These findings align with recent literature emphasizing the role of hybrid representation learning in clinical diagnostics. The integration of SHAP-based interpretability further supports the model’s utility in clinical decision support, offering transparency alongside performance. The architecture remains computationally efficient, achieving low inference latency suitable for real-time applications.

The framework is designed for practical deployment beyond experimental evaluation. With approximately 95 M parameters in the encoder and less than 2 M in the classifier, it performs real-time inference on standard CPUs with an average latency of 75.9 ms per sample. Its modular design allows the pre-trained encoder to be reused across devices or integrated into telemedicine platforms for remote voice-based screening. Automated preprocessing, normalization, and SHAP-based interpretability further support transparent, low-cost implementation in clinical or home settings, ensuring that the system is computationally efficient, scalable, and clinically interpretable.

## 7. Conclusions and Future Work

This paper proposed a multi-modal hybrid learning framework for early Parkinson’s detection by integrating structured voice biomarkers from the UCI Parkinson’s dataset with contrastive speech embeddings extracted from the DAIC-WOZ corpus. The model employs an early fusion strategy followed by a dense neural classifier, enabling the capture of both low-level acoustic irregularities and high-level affective patterns. Experimental results demonstrate that the proposed method consistently outperforms unimodal baselines and traditional fusion strategies, achieving a peak accuracy of 96.2% and an AUC of 97.1%. SHAP-based interpretability analysis further reveals the dominant contribution of select biomarkers and embedding dimensions, providing valuable insights for clinical application.

Future work will extend the current framework in several directions. First, temporal modeling using recurrent or transformer-based encoders may enhance the sensitivity to speech dynamics over time. Second, we plan to expand the dataset scope to include multilingual and noisy real-world speech samples to test cross-lingual generalization and robustness. Third, deployment of the model in a mobile health environment is envisioned, supported by real-time inference and lightweight on-device computation. Finally, longitudinal tracking of subjects using sequential voice recordings could support disease progression monitoring and early intervention planning.

## Figures and Tables

**Table 1:** Systematic comparison of Parkinson’s disease diagnostic frameworks highlighting trade-offs in accuracy, interpretability, and real-world deployability.

Ref.	Modality	Feature Type	Fusion Strategy	Interpretability	Performance	Deployment Feasibility	Limitations
[27]	Voice only	Handcrafted features	None (unimodal)	Moderate (statistical tests)	AUC: 65.9%	High (lightweight)	Low performance; does not handle subtle depressive cues
[28]	Voice only	Handcrafted + PCA	None (unimodal)	High (feature-level)	Accuracy: 94% (RF)	Moderate	No deep semantic features; moderate scalability
[29]	Voice only	CNN (xDMFCCs)	None (unimodal)	High (explainable DL)	F1-score: 75%	High	Task mismatch (brain lesions); not optimized for PD
[33]	Audio + Visual	Disentangled embeddings	Early dual-stream	Low	High speaker ID accuracy	Low (video + audio required)	Not diagnostic; lacks clinical relevance
[34]	Voice only	SSL embeddings (Wav2Vec 2.0)	None (unimodal)	Low	WER: 1.8%/3.3%	High	Not interpretable; not PD-specific
[35]	Voice only	SSL + attention	None (unimodal)	Moderate–High	Competitive PD accuracy	High	No structured feature integration
[36]	Voice only	SSL + paralinguistic cues	Multi-stage pipeline	Moderate (emotion alignment)	+2.9% over baselines	Medium	Task-focused on dialogue, not disease classification
[37]	Motion + Audio + Facial	Encoded RGB + sparse-coded features	STN + RGA	Low	Strong multi-task performance	Low (sensor-rich setup)	Complex imaging required; not voice-centric
[38]	T1WI + DTI + Clinical	Structured imaging + early assessment	Stacking ensemble	Low	Accuracy: 96.88%	Low (requires neuroimaging)	Expensive and non-scalable; lacks transparency
[39]	MRI + PET	CNN-learned image fusion	Multi-focus CNN	Low	Accuracy: 97.19% (DenseNet)	Low (heavy imaging)	Not voice-based; lacks real-time feasibility
[40]	MRI + Gait + Speech	Hierarchical attention + optimization	Hierarchical attention	Moderate (SHAP-CAM)	Accuracy: 94.2%	Low (multi-modal sensors)	High model complexity; sensor overhead
[24]	EEG + EMG + ACC + SC	Time, frequency- domain features	Sensor-level early fusion	Moderate	F1-score: 99.19%	Low (wearable dependent)	High accuracy but hardware-heavy and non-scalable
Proposed	Voice (UCI + DAIC-WOZ)	Biomarkers + Wav2Vec2 embeddings	Early modality-aware fusion	High (SHAP + ablation)	Accuracy: 96.2%, AUC: 97.1%	High (voice-only, real-time ready)	Small dataset; further cross-lingual validation required

**Table 2:** Comparative Performance Across Models.

Model	Accuracy	F1 Score	AUC
UCI Voice Biomarkers Only	89.5%	0.88	0.91
DAIC-WOZ Embeddings Only	91.7%	0.90	0.93
Early Fusion (Concat)	93.4%	0.92	0.95
Late Fusion (Voting)	92.1%	0.91	0.94
Proposed Hybrid Classifier	96.2%	0.95	0.971

## Data Availability

No new data were created or analyzed in this study.
